# Oocyte maturation, fertilization, and embryo development in vitro by green and chemical iron oxide nanoparticles: a comparative study

**DOI:** 10.1038/s41598-024-65121-1

**Published:** 2024-06-19

**Authors:** Shamim Nejadali Chaleshtari, Elaheh Amini, Farzaneh Baniasadi, Somayeh Tavana, Mohammadreza Ghalamboran

**Affiliations:** 1 Department of Animal Biology, Faculty of Biological Sciences, Kharazmi University, Tehran, Iran; 2 Department of Embryology, Reproductive Biomedicine Research Center, Royan Institute for Reproductive Biomedicine, ACECR, Tehran, Iran; 3 Department of Plants Sciences and Biotechnology, Faculty of Life Sciences and Biotechnology, Shahid Beheshti University, Tehran, Iran

**Keywords:** In vitro maturation, Green synthesis, Iron oxide nanoparticles, Antioxidants, Mouse oocyte, Biotechnology, Medical research, Nanoscience and technology

## Abstract

Oxidative stress is considered one of the main challenges for in vitro maturation (IVM) and makes assisted reproductive technology (ART), including IVF and embryonic development less effective. Reducing free radicals via biocompatible nanoparticles (NPs) is one of the most promising approaches for developing IVM. We investigated the comparative effect of green and chemically synthesized iron oxide nanoparticles (IONPs) with an aqueous extract of date palm pollen (DPP) on oocyte parameters related to the IVM process. To this end, IONPs were synthesized by chemical (Ch-IONPs) and green methods (G-IONPs using DPP) and characterized. The mature oocyte quality of the Ch-IONPs and G-IONPs groups was evaluated by JC1 and Hoechst staining, Annexin V-FITC-Propidium Iodide, 2′, 7′-dichlorofluorescein diacetate, and dihydroethidium staining compared to the control group. Eventually, the mature oocytes were fertilized, promoted to blastocysts (BL), and evaluated in vitro. Compared with the control and G-IONPs groups, the Ch-IONPs-treated group produced more hydrogen peroxide and oxygen radicals. Compared with the Ch-IONPs group, the fertilization rate in the G-IONPs and control groups increased significantly. Finally, the G-IONPs and control groups exhibited a significant increase in the 2PN, 2-cell, 4-cell, 8-cell, compacted morula (CM), and BL rates compared with the Ch-IONPs group. Green synthesis of IONPs can reduce the toxicity of chemical IONPs during the IVM process. It can be concluded that G-IONPs encased with DPP compounds have the potential to protect against exogenous reactive oxygen species (ROS) production in an IVM medium, which can have a crucial effect on oocyte maturation and fertilization efficiency.

## Introduction

Researchers have reported the prevalence of infertility as a male or female reproductive disease in approximately 50–70 million couples worldwide in recent decades^1^. Male factor infertility contributes to approximately 30–50% of infertility and testicular dysfunction; endocrine pathology, lifestyle, and congenital factors can also be detrimental factors in its prevalence^2^. Female infertility can be attributed to various factors, including endometriosis, uterine abnormalities, obstructed fallopian tubes, and ovulation disorders. Among these, polycystic ovarian syndrome (PCOS) stands out as a prevalent example, characterized by a diverse range of female endocrine disorders. Assisted reproductive technology (ART) can serve as a valuable strategy for treating PCOS^3^. Gonadotropin therapy is a treatment option for patients with PCOS. However, to increase the number of oocytes in in vitro fertilization (IVF), which is one of the ART procedures, ovarian stimulation is required. However, this method increases the risk of ovarian hyperstimulation syndrome (OHSS). Therefore, the IVM strategy is a prerequisite for reducing the risk of OHSS because extracting immature oocytes from antral follicles doesn’t require stimulating oocytes; they would be matured in vitro and then fertilized with the IVF process^4^. Nevertheless, the elevation of intracellular ROS in IVM cultivation medium is a major challenge that disturbs this process. To overcome this problem, the use of a radical-scavenger supplement is required. Several studies have demonstrated that adding supplements, such as herbal antioxidants, to the human oocyte IVM medium can improve its efficacy^5,6^.

Recently, researchers proposed considering nanotechnology to overcome impairments in reproductive medicine. Studies have reported that some nanoparticles (NPs) exhibit antioxidant and antibacterial activities. NPs also positively affected oocyte quality, embryo cleavage, and blastocyst rate^7^. According to Abdel-Halim et al., chitosan nanoparticles can inhibit the harmful effects of linoleic acid on the growth and development of bovine oocytes^8^. Tiedemann et al. have also demonstrated that the simultaneous exposure of pig oocytes to gold nanoparticles coated with bovine serum albumin (BSA) did not exhibit any detrimental effects on the maturation process of the oocytes^9^.

The manipulation of NPs can potentially decrease ROS levels, as evidenced by their ability to mitigate oxidative stress and ROS elimination^10,11^. Metal nanoparticles, such as IONPs, have gained particular interest in biomedical applications due to their biocompatibility, chemical stability, superparamagnetic properties, and low cost^12^. However, research has shown that IONPs may increase ROS production when not attached to other substances. The synthesis method of IONPs can also influence their toxicity and biocompatibility^13^. Although most chemical synthesis methods require less synthesis time, the most prominent ones are costly, consume high energy, utilize dangerous chemicals, and encounter issues with their physicochemical properties and biocompatibility. Conversely, it revealed that green synthesis methods using plant or agro-waste extracts can reduce the toxicity of NPs and make them biocompatible and cost-effective. In this way, bioactive materials taken from plants using capping and reducing agents have advantages such as preventing particles from sticking together and making them more stable during synthesis^14,15^.

Phoenix dactyliferous L. is a flowering plant species in the palm family, Aceraceae, commonly known as date palm. This plant has various biological and medicinal properties, including antimicrobial, anti-oxidative, anti-toxicant, and anti-inflammatory. Date palm pollen (DPP) is the male reproductive dust from palm flowers, which can enhance fertility rates in both men and women. It benefits human reproductive health because of its adequate nutrients, such as vitamins, minerals, and other organic nutrients^16–18^. Studies have shown that catechin, coumarin, and caffeic acid are bioactive components of DPP, and possess strong antioxidant properties. For its parts like sterols, steroidal saponin glycosides, estrone-like compounds, and estrogen-like compounds^17^, DPP seems to play a big part in making women more fertile.

The presence of bioactive compounds in DPP for reducing and capping NPs has previously been proven in the synthesis of gold and silver nanoparticles^19^. Thus, this study has been conducted to synthesize IONP by chemical and green methods using DPP extract and evaluate the effect of synthesized IONPs on the in vitro maturation rate of mouse GV oocytes, ROS level, and fertilization rate of oocytes. Here, we synthesized IONP nanoparticles through two strategies: (A) the chemical co-precipitation method and (B) the green modified co-precipitation method using DPP aqueous extract as a biological source of reduction agent. Biological assays related to mouse GV oocyte maturation.

## Material and methods

All chemical materials and kits were purchased from Sigma-Aldrich, USA. Otherwise, the producer companies were addressed. The study received approval from the Medical Ethics Committee of Kharazmi University (IR.KHU.REC.1401.007) to conduct the experimental procedures. All experiments were performed following the relevant guidelines and regulations. Moreover, all methods were reported following ARRIVE guidelines (https://arriveguidelines.org) for reporting animal experiments.

### Preparation of DPP aqueous extract

DPP was collected from Bushehr province and deposited at the Herbarium of the Plant Department, Kharazmi University, Tehran, Iran (specimen voucher number: 25556). The male reproductive organs of the date palm are dried and stored at a temperature of 25 °C to obtain DPP. 1 g of powdered DPP was introduced into 99 ml of distilled water. Following a 1-h incubation at 35 °C, the mixture underwent 2 h of agitation. Three rounds of centrifugation were performed on the samples at 4000 rpm for 20 min. Subsequently, it was passed through a mesh filter and a filter with a pore size of 0.45 µm. The collected liquid portion was stored at a temperature of 4 °C for subsequent utilization^20^.

### Synthesis of iron oxide nanoparticles and their characterization

The G-IONPs were synthesized using the modified Yusefi et al. protocol in 2021^20^. 2.53 gr of FeCl3. 6H2O and 0.99 gr of FeCl2⋅4H2O were each added to 100 ml of DPP aqueous extract in a molar ratio of 2:1. After 30 min of vigorous stirring, the pH of each solution was adjusted to 11 by slowly adding 1 M NaOH. Finally, the obtained precipitates were oven-dried at 50 °C after being centrifuged three times at 14,000 rpm for 12 min. The same method with more NaOH was used to create bare Fe3O4 NPs (Ch-IONPs) without the extract.

An X-ray diffractometer (Rigaku D-max CIII) was used to analyze the IONPs, and Fourier transform infrared (FT-IR) characterized the functional groups of the samples (Perkin-Elmer-RXI FT-IR spectrophotometer) with KBr pellets. The surface charge of IONPs was determined using a zeta potential assay (Zetasizer Microtrac Wavell). A field emission scanning electron microscopy (FESEM) (TESCAN-MIRA III-FEG) was used to describe the size, shape, and polydispersity of the synthesized IONPs. To assess the purity of the Fe3O4 elements and to detect the presence of elements from organic moieties present in the DPP extract, we employed energy-dispersive X-ray spectroscopy (EDX) to determine the elemental composition of the produced IONPs.

### Group design

Naval Medical Research Institute (NMRI) mice were sacrificed by dislocating their cervical vertebrae and were used in this investigation to collect denuded GV oocytes. The mice were 6–8 weeks old. The Royan Research Institute's animal lab housed the animals under typical conditions, including a temperature range of 18–25 °C, humidity levels ranging from 40 to 60%, a periodic light–dark cycle of 12.12 h, and an abundance of food and drink. This study examined three groups: (1) a control group that did not get any IONP exposure; (2) a Ch-IONPs group that received chemically synthesized IONPs; and (3) a G-IONPs group that received green IONPs composed of DPP extract.

### In vitro maturation of oocytes

Using a stereomicroscope (SMZ 800; Nikon, Tokyo, Japan), the GV oocytes were collected from the ovaries and transplanted to a dissected medium. In IVM medium, which includes Alpha Minimum Essential Medium (α-MEM; Gibco, USA), 7.5 IU/ml human chorionic gonadotropin (hCG; Choriomon, Switzerland), and 0.01 IU/ml Follicle Stimulating Hormone (FSH; Merck, Germany), an oocyte and its surrounding cumulus cells were transferred. Mineral oil was then applied as the final touch. An inverted microscope was used to determine the rates of germinal vesicle breakdown (GVBD), metaphase II (MII), and GV arrest after 18 h of incubation at 37 °C with 5% CO_2_, and 95% humidity.

### Toxicity test

To obtain the optimal concentration of synthesized IONPs in the oocyte maturation culture medium, the oocyte maturation rate and mitochondrial membrane potential were compared. The IVM rate was assessed 18 h after incubating the oocyte in an IVM medium containing different concentrations of IONPs (0, 1, 2, and 4 μg/ml) to find the most effective dose. After that, JC1 was carried out to evaluate Mt activity.

### Mitochondrial distribution and membrane potential

The Mt membrane potential was evaluated using the JC-1 staining technique, which involved the utilization of 5, 50, 6, 60-tetrachloro-1, 10, 3, 30 -tetraethylbenzimidazolylcarbocyanine iodide dye. Incubate the MII oocytes in α-MEM medium with 10% FBS and 2 mM JC-1 for 30 min. The oocytes were then scrutinized using fluorescent e (LSCM, Leica, Germany). Mitochondria exhibiting an inner membrane potential below 100 mV and exceeding 140 mV emitted green and red fluorescence, respectively. The red-to-green light ratio was utilized to ascertain Mt activity. We quantified the red and green signals using Image J software (Image J 1.46r, Java 1.6.0_20).

### Determination of apoptosis rate in oocytes

Annexin-V-FITC binds to phosphatidylserine in the cell membrane, which is an apoptosis induction marker. Staining with propidium iodide (PI) can segregate live cells from dead cells. In this method, viable oocytes revealed negative Annexin-V and PI signals; early apoptotic oocytes showed an Annexin-V positive signal and a PI negative signal; late apoptosis showed positive Annexin-V and PI signals; and necrotic oocytes exhibited a negative Annexin-V and positive PI signal.

Here, an Anx_PI kit from IQ-Products in the Netherlands was used to assess oocyte viability. Following a 20-min incubation of the MII oocytes in drops of Annexin V on ice, samples were supplemented with PI at a concentration of 4% (v/v). Subsequently, the samples were scrutinized for apoptosis and necrosis using fluorescence microscopy. Annexin V emitted a green signal upon activation of the fluorescent light, whereas PI emitted a red signal. The oocytes were categorized into four groups: alive and non-apoptotic oocytes (Anx⁻_PI⁻), early apoptosis oocytes (Anx⁺_PI⁻), necrotic oocytes (Anx⁻_PI⁺), and dead or late apoptosis oocytes (Anx⁺_PI⁺)^21^.

### Detection of oocyte maturation

Oocyte nuclear maturation after IVM was assessed by Hoechst 33248 staining. The oocytes were stained by adding 10 µl of dye at 1 mg/ml concentration to 1 ml of media. After 15 min, a fluorescent microscope (with an excitation wavelength of 330–385 nm) was used to detect whether the DNA belonged to the polar body, and the nucleus was stained^21^.

### Evaluation of the intracellular and extracellular oocyte ROS

The intracellular ROS levels in the oocytes were assessed using 2′, 7′-dichlorofluorescein diacetate (DCFH-DA), and dihydroethidium (DHE) dyes at doses of 10 µM and 5 µM, respectively. Oocytes were placed in 50 µl drops of DCFH-DA and DHE solutions and left to incubate at 37 °C, 5% CO_2_, and 95% humidity for 30 min. The oocytes were then washed with 10 µl drops of serum-free medium. A fluorescence microscope then detected ROS levels in the DCFH-DA and DHE experiments at 610 and 590 nm, respectively.

For extracellular ROS, IVM media belonging to the control, Ch-IONPs, and G-IONPs were collected. To this end, DCFH-DA was added to the oocyte culture medium at the same volume to obtain a 10 µM stain concentration. This experiment examined ROS in the culture medium by converting a dye into fluorescent dichlorofluorescein at a wavelength of 480 nm and a fluorescence emission intensity of 520 nm^22^.

### In vitro fertilization and embryo development

To induce sperm capacitation, the epididymides were extracted from an 8-week-old male mouse and incubated in 1 ml of T6 medium supplemented with 15 mg/ml of BSA for 1 h. Add 2 µl of capacitated sperm to 50 µl of IVF medium (T6 contains 15 mg/ml of BSA droplets coated with mineral oil), including 5 oocytes. They were placed in an incubator with 5% CO_2_ at 37 °C. Six to eight hours after insemination, we detected two pronuclei (2PN)-stage zygotes under an inverted microscope (Olympus, Japan). Development medium (SAGE; ORIGIO, Denmark) was subsequently applied for embryonic development in zygotes. The rates of 2-cell, 4-cell, 8-cell, CM, and BL were recorded for five days after insemination^23^.

### Scoring the number of cells in blastocysts

DAPI staining was used to assess cell deviation (Salgado et al. 2018). After placing the blastocysts in drops containing 10 µg/ml DAPI, they were incubated in the dark for 30 min. Afterward, they underwent washing in α-MEM medium. Fluorescent images were obtained using a fluorescence microscope at a wavelength of 330–385 nm.

### Statistical analysis

Statistical analysis was conducted using the GraphPad Prism software version 9. The ANOVA (analysis of variance) and Kruskal–Wallis tests are used to analyze variances for normal and non-normal distributions, respectively. The results were shown as the mean plus or minus the standard error of the mean (mean ± SEM). The differences shown by asterisks are considered significant (*P < 0.05, **P < 0.01, ***P < 0.001, and ****P < 0.0001). As the ns implies, the results are not statistically significant.

### Ethics approval and consent to participate

The researchers followed the guidelines provided by the Medical Ethics Committee of Kharazmi University (IR.KHU.REC.1401.007) to conduct the experimental procedures.

## Results

### Characterization of IONPs synthesized by chemical and green methods

#### X-ray diffraction (XRD) pattern

**Figure 1:**
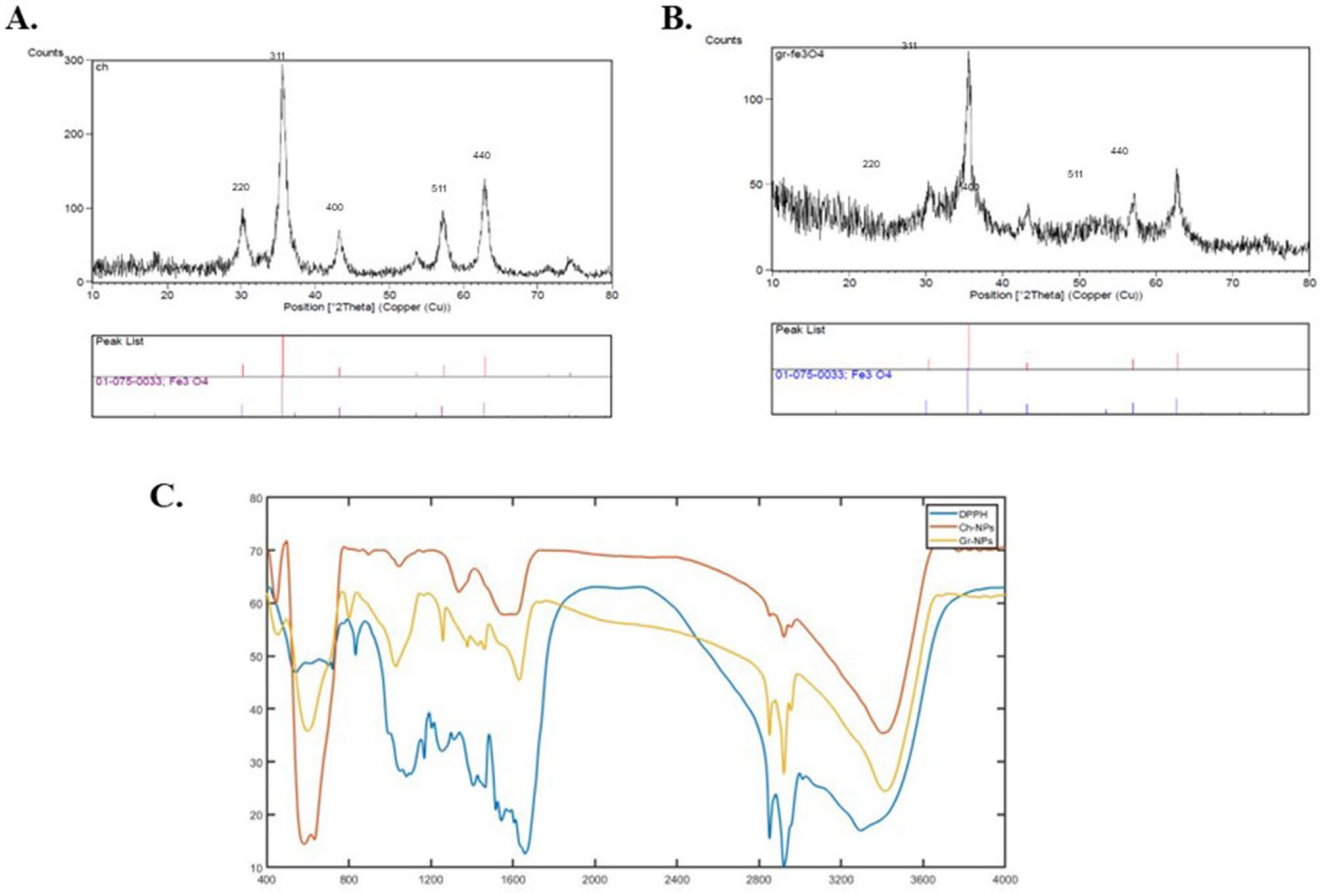
Fe3O4 NPs were characterized using powder XRD to determine the purity and crystal nature of the NPs. (**A**) Ch-IONPs; (**B**) G-IONPs synthesized using DPP extract. (**C**) FTIR analysis of Ch-IONPs and G-IONPs for determining the functional group of the NPs. *G-IONPs* green synthetized iron oxide nanoparticles, *Ch-IONPs* chemically synthetized iron oxide nanoparticles.

Nanocrystals were found in the powdered samples, and the XRD analysis revealed any changes in the chemicals that made up the IONPs made with the green method (DPP extract). Figure 1 shows the XRD patterns for IONPs made using the chemical and green methods based on Miller's indexing criteria. The six peaks in the 2θ angles are located at 30.5, 35.7, 43.2, 53.7, 57.4, and 63 °C. Based on Miller's indexing criterion, these peaks correspond to Miller indices 220, 311, 400, 422, 511, and 440. The alignment of the peaks of Ch-IONPs and the green sample, which were produced using 1 g of the extract, exhibited a minor variation in the θ angle, suggesting the formation of G-IONPs. Based on the data acquired from the XRD examination and using Scherer's equation (Fig. 1A,B), we determined that the size of the IONPs produced using the chemical and green approaches was 51.9 and 6.22 nm, respectively.

#### FTIR analysis

FT-IR analysis was conducted to confirm the synthesis of IONPs using DPP extract and the creation of a covalent link between the DPP extract and the iron oxide core. The FTIR spectra of DPP extract and nanoparticles made with DPP extract using both chemical and green methods are shown in Fig. 1C. The spectra exhibited a prominent peak within the range of 3250–3500 cm^−1^, which corresponds to the hydroxyl groups found in the structure of both the DPP extract and G-IONPs. There are three peaks commonly observed at frequencies of 1465, 1259, and 1163, which correspond to the C–H and C–O groups. These peaks are present in both the DPP extract and the G-IONPs synthesized using the DPP extract. The spectra of all three types of IONPs created using DPP extract and Ch-IONPs exhibit a distinct peak within the 580–630 cm^−1^ wavelength range. This peak corresponds to the Fe3O_4_ nanoparticles and is attributed to the Fe–O group. Its presence serves as evidence that the IONPs were indeed synthesized.

#### Zeta potential assay

The results of the zeta potential test allude to different charges of Ch-IONPs and G-IONPs at + 0.2 mv and − 0.4 mv, respectively. Because DPP contains lipid molecules with a negative charge, when these molecules bind to the surfaces of nanoparticles during the nanoparticle synthesis process, the nanoparticles' electric charge changes to a negative one. This alters the distribution and balance of the electric charge on the oocyte surface.

#### FESEM and EDX analysis

**Figure 2:**
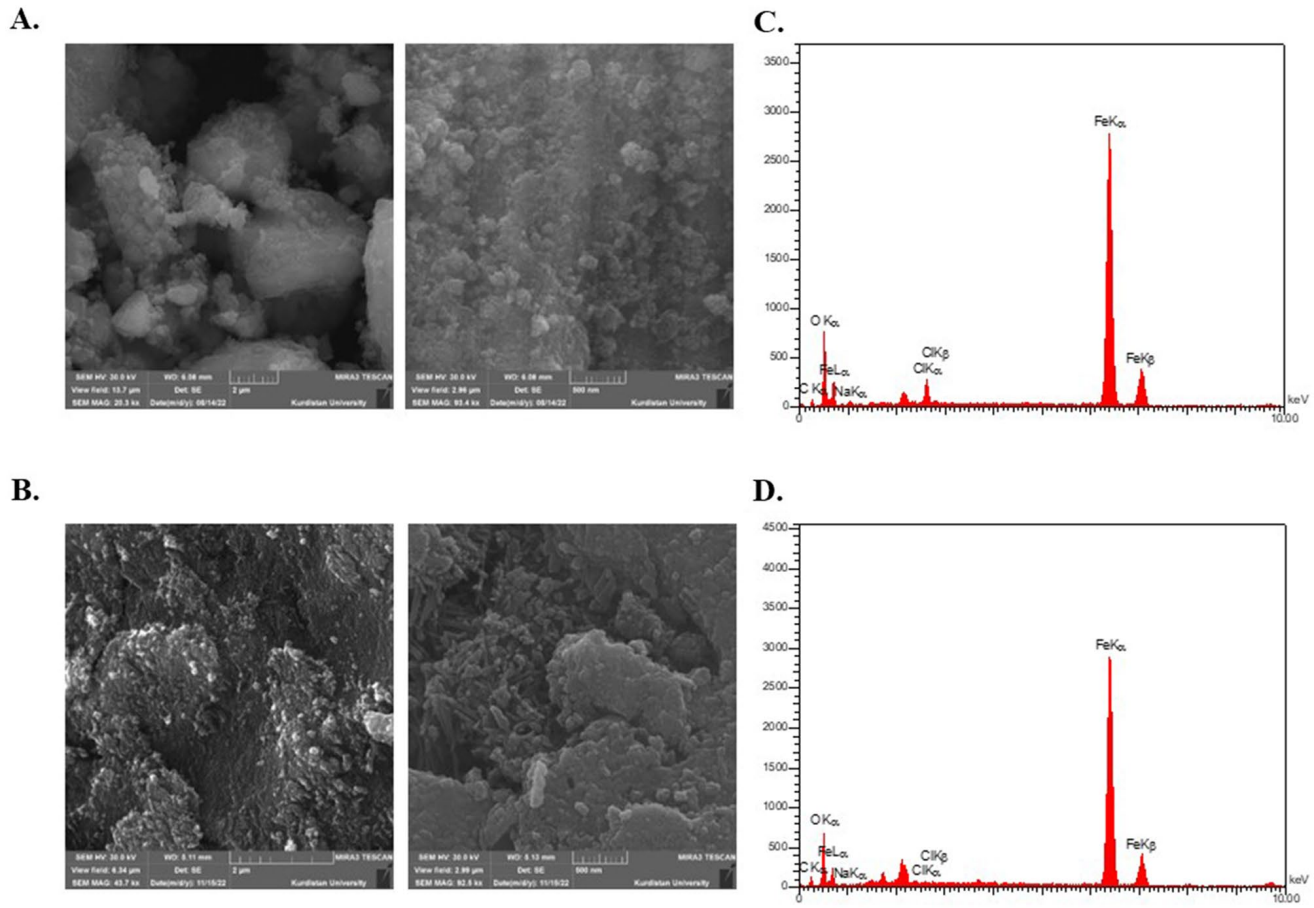
FESEM and EDX elemental spectra of the Fe3O4 synthesized NPs by chemical and green methods. FESEM image of Fe3O4 nanoparticles at different magnifications. (**A**) Ch-IONPs and (**B**) G-IONPs; EDX elemental spectrum; (**C**) Ch-IONPs and (**D**) G-IONPs. *G-IONPs* green synthetized iron oxide nanoparticles, *Ch-IONPs* chemically synthetized iron oxide nanoparticles.

Figure 2A,B show that the FESEM exhibited a spherical morphology for Ch-IONPs and a combination of spherical and rod morphology for G-IONPs, respectively. It also seems that the particle size was less than 100 nm. Furthermore, the chemical nature of particles was examined by EDX, which is presented in Fig. 2C (Ch-IONPs) and 2D (G-IONPs). The EDX spectrum analysis confirms the presence of Fe and O elements with Fe3O4 stoichiometry.

#### 2,2-diphenyl-1-picrylhydrazyl (DPPH) assay

**Figure 3:**
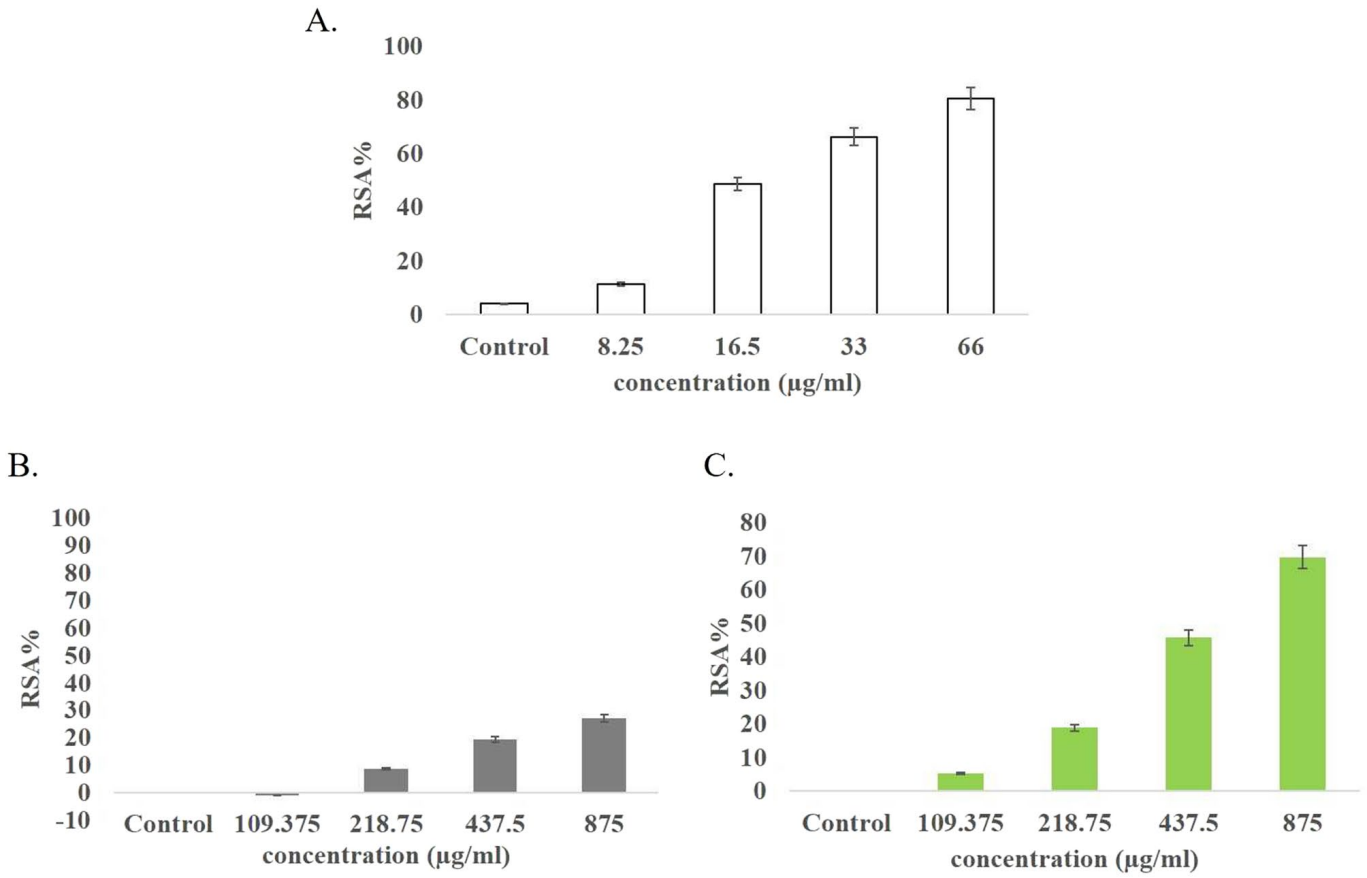
A DPPH test assessed the antioxidant activity of IONPs made from DPP extract and bare Fe3O4. In this assay, vitamin C serves as the standard. (**A**) Vitamin C; (**B**) Ch-IONPs; and (**C**) G-IONPs. *G-IONPs* green synthetized iron oxide nanoparticles, *Ch-IONPs* chemically synthetized iron oxide nanoparticles.

Figure 3 displays the chart depicting the DPPH assay results for Ch-IONPs, G-IONPs, and DPP extract. Vitamin C, a widely recognized antioxidant molecule, is typically compared with the outcome of this assay. The ROS scavenging activity percentage was measured in two graphs. The results showed that G-IONPs made from the DPP extract had an antioxidant effect of about 70%. On the other hand, Ch-IONPs show a reduction of around 30% in free radical inhibitory activity.

#### Toxicity assay for Ch-IONPs

**Figure 4:**
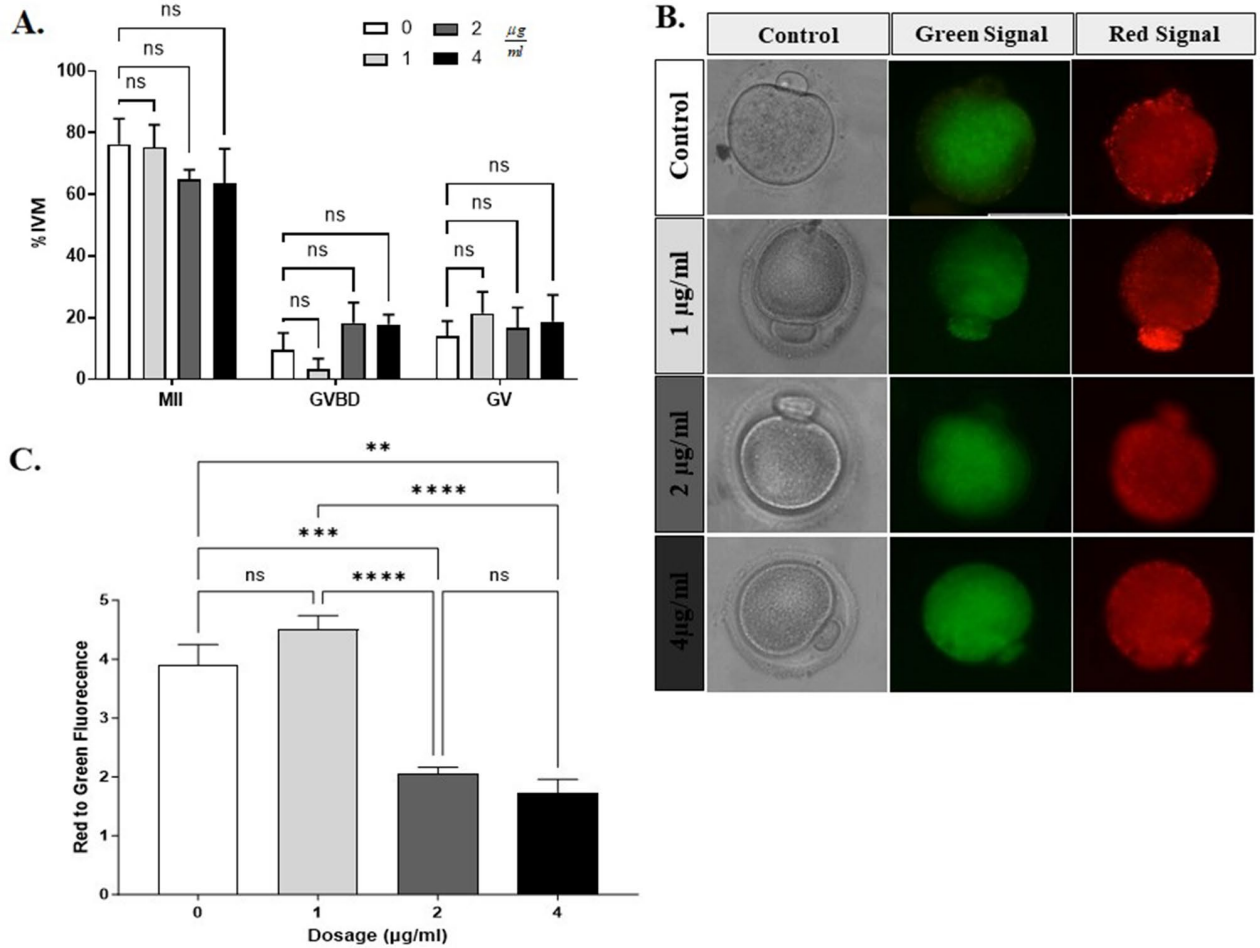
Toxicity analysis based on maturation rate and Mt membrane potential. (**A**) Rates of MII, GV, and GVBD oocytes at different concentrations of Ch-IONP (data presented as mean ± SEM, n = 5). (**B**) Red/green fluorescence signal in MII oocytes; (**C**) Mt activity rates (mean ± SEM) in 0, 1, 2, and 4 µg/ml Ch-IONP groups. *Ch-IONPs* chemically synthesized iron oxide nanoparticles.

Six replications were considered for the IVM process in the toxicity test. To this end, 329 GV oocytes were obtained from 10 NMRI female mice and randomly allocated to four groups. The groups included a control group and experimental groups exposed to three distinct doses of Ch-IONPs (1, 2, and 4 µg/ml). Figure 4A illustrates the outcomes of the IVM process. Figure 4A demonstrates that increasing the concentration of Ch-IONPs leads to a reduction in the MII rate. No significant difference was observed between the control group i.e. 0 µg/ml (76.31 ± 8.22) and the groups treated with 1 µg/ml (70.36 ± 7.75), 2 µg/ml (59.71 ± 5.80), and 4 µg/ml (69.60 ± 11.02) of the substance. However, the group that received 1 µg/ml Ch-IONP showed the highest maturation rate compared to the others.

In terms of IVM rates, no statistical differences were observed; the JC1 staining had been completed to determine the non-toxic dosage. To conduct the JC1 assay for toxicity testing, 3–5 MII oocytes were selected per replication, and group. Figure 4B displays the observed fluorescence signal of Mt membrane potential in MII oocytes derived from GV oocytes treated with varying doses of Ch-IONP. Figure 4C then displays the data from the red-to-green signal analysis. The five statistical replications were used to present meaningful data between groups. However, the Mt membrane potential was much higher in the 1 µg/ml Ch-IONPs group (4.50 ± 0.28) compared to 2 µg/ml (2.05 ± 0.11) and 4 µg/ml (1.72 ± 0.23) groups (*p < 0.05). It was also determined that this value was insignificant compared to the MII oocytes in the control group (3.89 ± 0.36). The results of the IVM rates and Mt activity determined the optimum concentration to be 1 µg/ml. Additionally, this dosage was utilized for the investigations involving Ch-IONPs and G-IONPs.

### Nuclear maturation, mitochondrial membrane potential, and viability under optimal IONP dosage

#### In vitro maturation analysis

**Figure 5:**
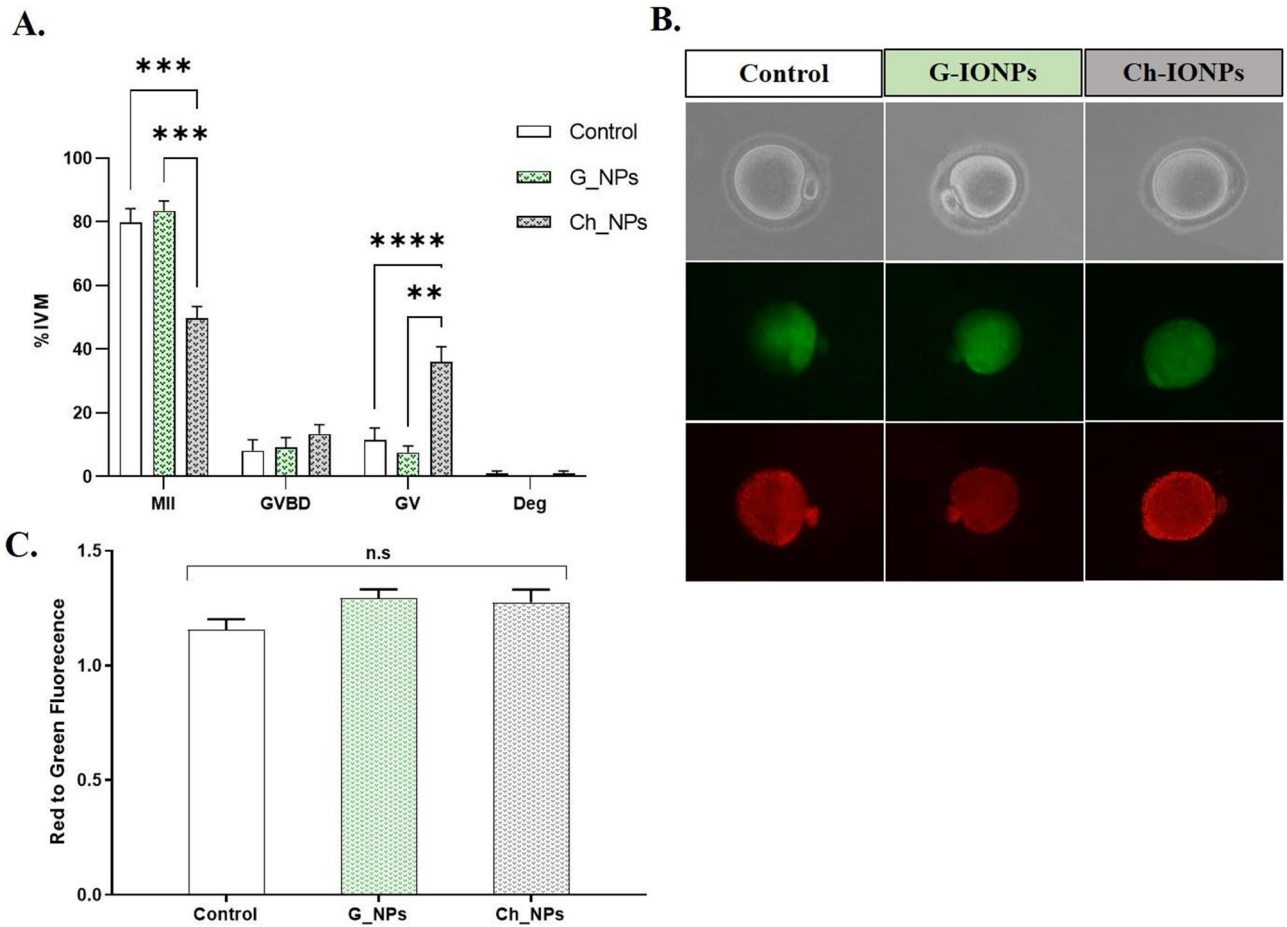
The MII oocyte quality analysis. (**A**) The rate of MII, GV, GVBD, and Deg oocytes in the Control, Ch-IONPs, and G-IONPs groups (****p < 0001, ***p < 0.001, **p < 0.01) (n = 5). (**B**) Mt membrane potential assessment for mouse MII oocytes in control, chemical, and green IONP groups. The data were shown with a mean ± SEM. ns means an insignificant difference. (**C**) The red-to-green fluorescence intensity and quantity (n = 5). *G-IONPs* green synthetized iron oxide nanoparticles, *Ch-IONPs* chemically synthetized iron oxide nanoparticles.

In this case, the IVM technique was carried out using a non-toxic dose of 1 µg/ml. This procedure involved using Ch-IONPs and G-IONPs, as well as a control group without IONPs. The experiment was replicated five times for statistical analysis. As shown in Fig. 5A, the MII rate is much lower in Ch-IONPs (49.80 ± 3.57) compared to the control (79.67 ± 4.49) and G-IONPs (83.48 ± 3.06) (***p < 0.001). The number of GV arrests in the Ch-IONPs group (36.03 ± 4.74) has meaningfully increased compared to the control (11.38 ± 3.84) (****p < 0.0001) and G-IONPs (7.50 ± 2.11) (**p < 0.01) groups.

#### JC1 staining

JC1 staining was performed on 3–5 MII oocytes from each control, G-IONPs, and Ch-IONPs group in each replication. Data were statistically analyzed in four replications. Figure 5B displays the fluorescent images in green and red. The mitochondria's membrane potential or activity belonging to MII oocytes went up in the IVM medium with 1 µg/ml Ch-IONPs (1.28 ± 0.06) and G-IONPs (1.29 ± 0.04) compared to the control (1.16 ± 0.05), however, the difference wasn't statistically significant, as shown in Fig. 5C.

#### Annexin V-PI staining

**Figure 6:**
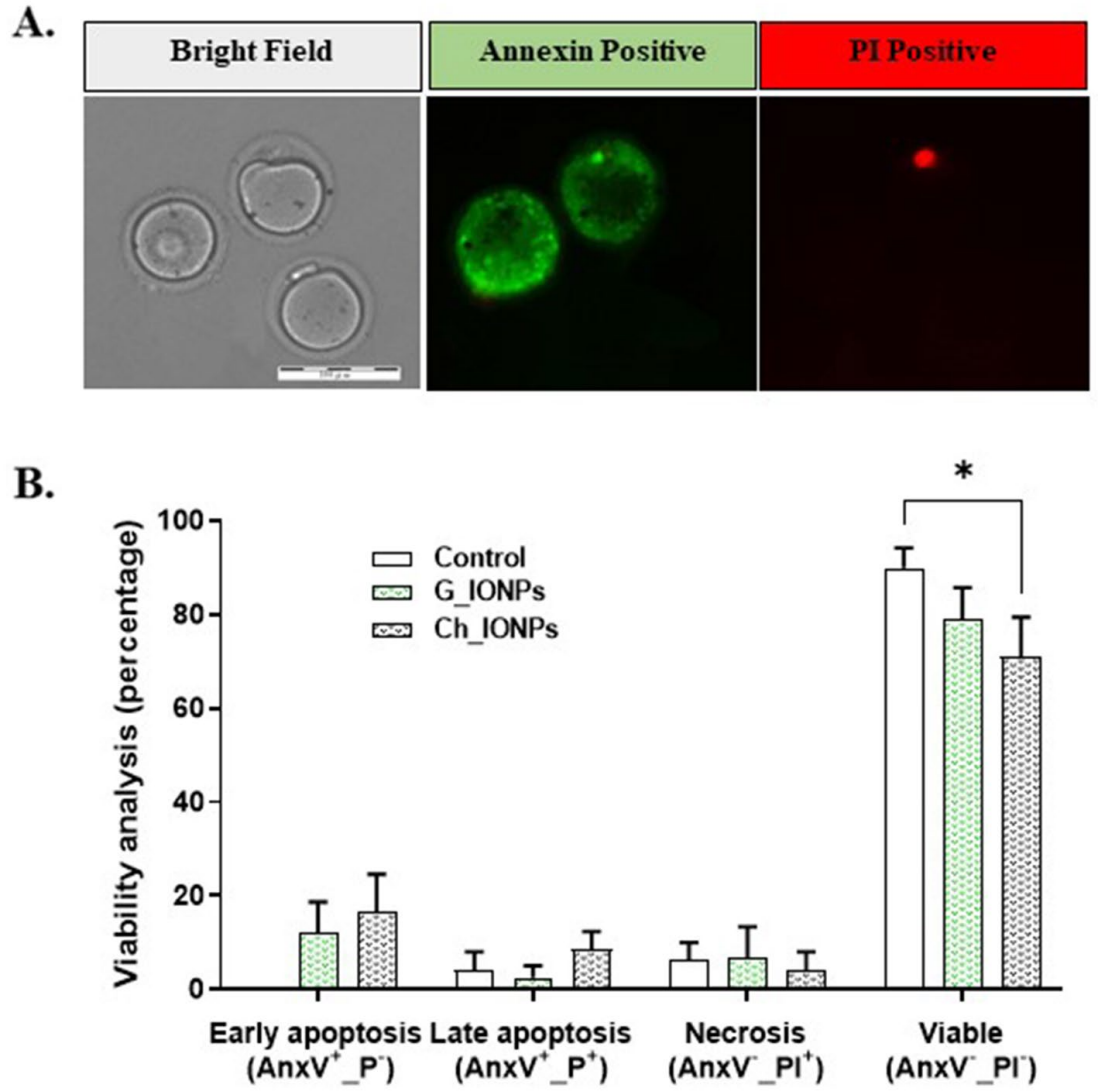
Annexin-V/PI double-staining in mouse MII oocytes in control, Ch-IONPs, and G-IONPs. (**A**) A bright field, a red signal in the nucleus (PI positive), and a green signal in the cytoplasmic membrane (Annexin-V positive). Scale bar 100 μm. (**B**) Quantification of viability analysis. Asterisks mean a significant difference (*p < 0.05). The ns indicates that it is not significant. *G-IONPs* green synthetized iron oxide nanoparticles, *Ch-IONPs* chemically synthetized iron oxide nanoparticles.

43 MII oocytes for each experimental group were stained using the Anx_PI kit. The mean ± SEM values of viability, apoptosis, and necrosis were conducted using five statistical analyses. Figure 6A shows a schematic of necrosis and early and late apoptosis. The control, G-IONPs, and Ch-IONPs show similar levels of apoptosis and necrosis (Fig. 6B). The oocyte viability rate was significantly lower in the Ch-IONPs group (71.00 ± 8.42) compared to the control group (89.86 ± 4.32) (*P < 0.05). In addition, the results showed no significant statistical difference between the control group and G-IONPs (78.84 ± 6.86).

#### Hoechst staining

**Figure 7:**
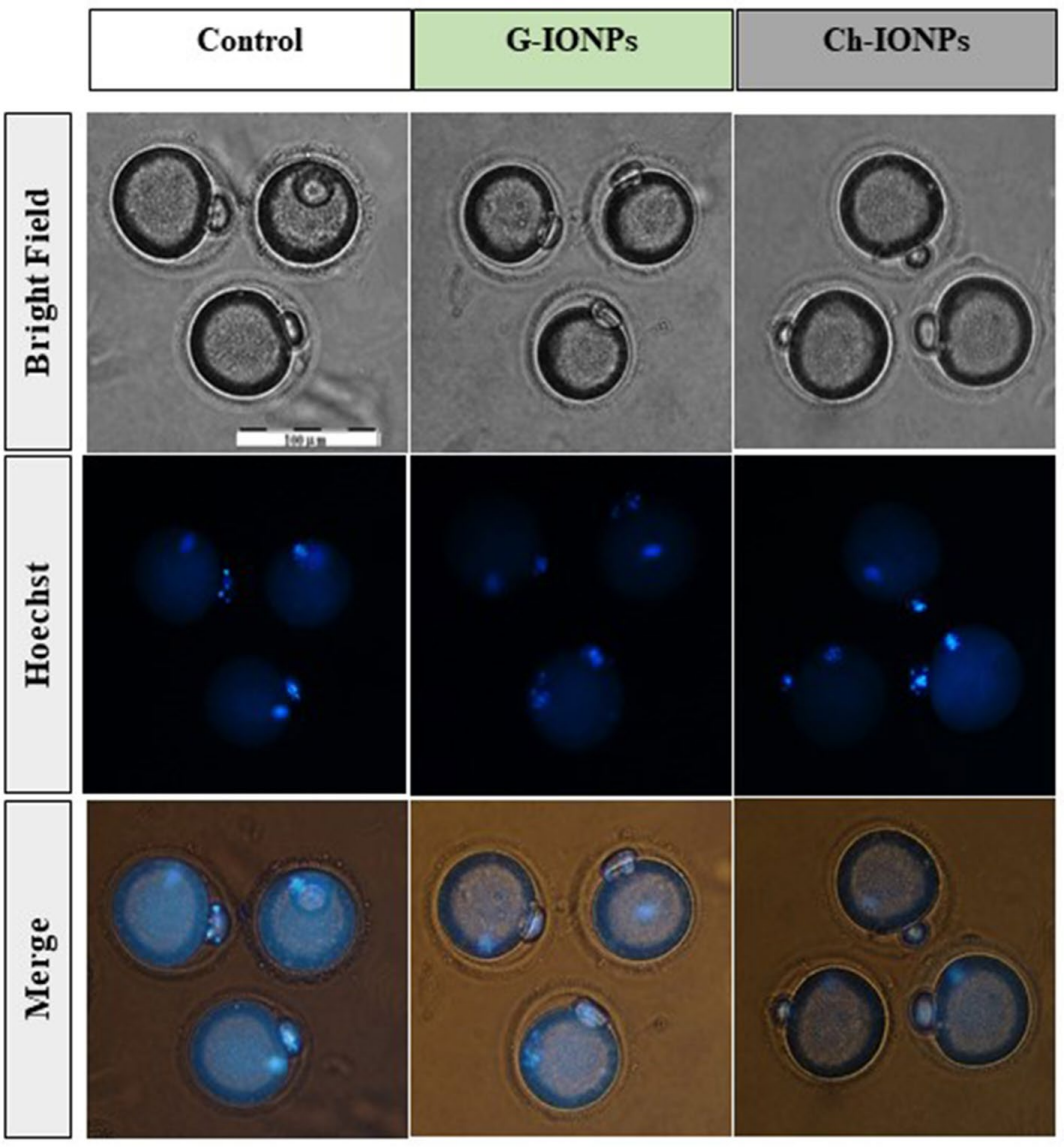
DNA staining of MII oocytes with Hoechst; bright field, fluorescent, and merge images; scale bar 100 μm.

Figure 7 exhibited the nuclear maturity of the mouse oocytes in the control, Ch-IONPs, and G-IONPs groups. The blue color of the nucleus in the oocyte and polar body indicates the validity of in vitro maturation of the mouse oocytes.

#### ROS evaluation by DCFH-DA and DHE

**Figure 8:**
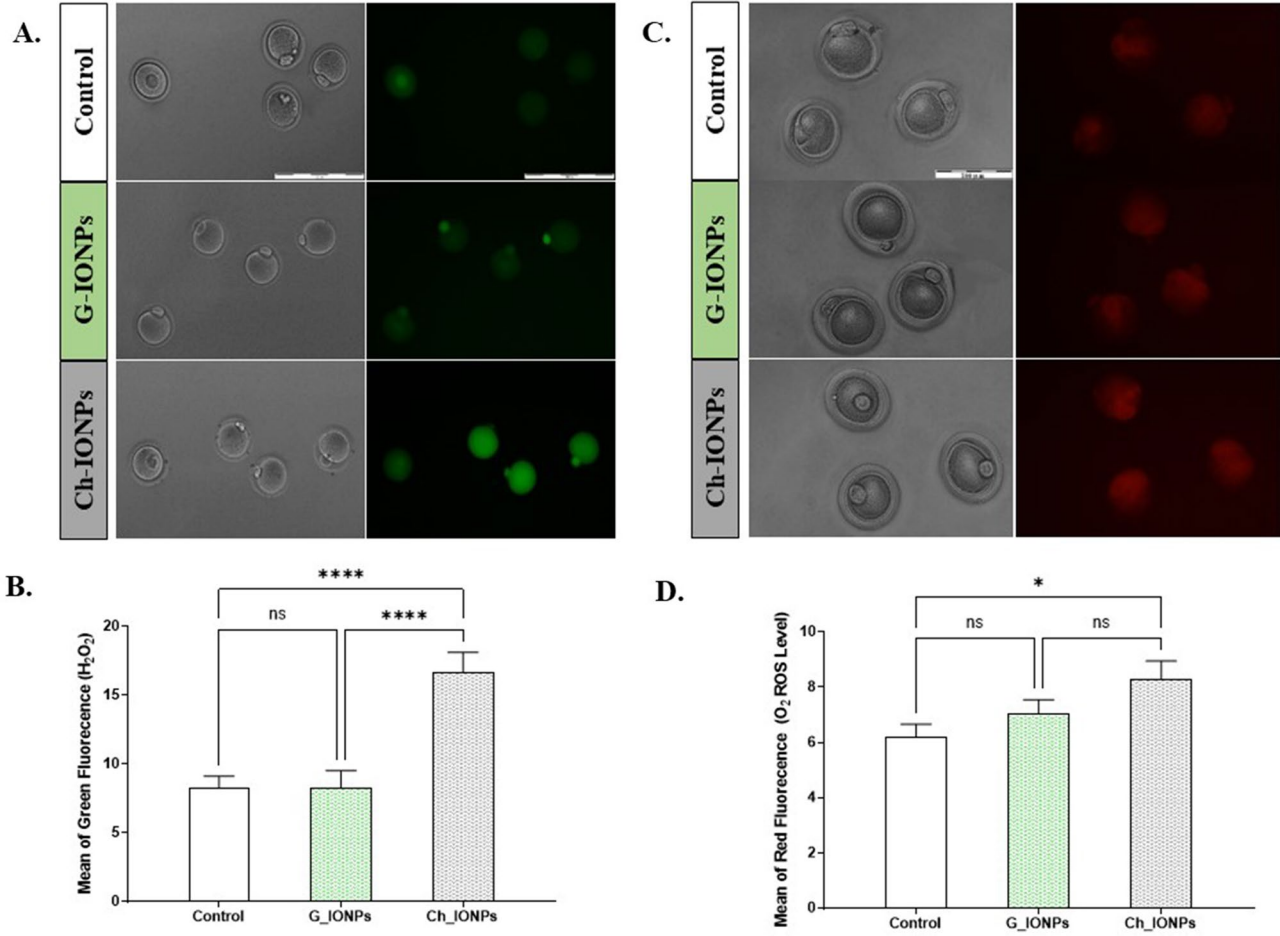
Intracellular ROS levels were evaluated with DCFH-DA and DHE in MII oocytes. (**A**) The mean fluorescence intensity of oocytes. (**B**) Representative photomicrographs of DCFH-DA staining. (**C**) The mean fluorescence intensity of oocytes. (**D**) Representative photomicrographs of DHE staining. (**E**) Extracellular ROS levels were assayed with DCFH-DA in the MII culture medium. The mean fluorescence intensity of the culture medium. Means ± SEM present the mean fluorescence intensity of the culture medium. Asterisks mean a significant difference (*p < 0.05, ****p < 0.0001). *DCFH-DA* 2′, 7′-dichlorofluorescein diacetate, *DHE* dihydroethidium.

DCFH and DHE staining, respectively, detected the hydrogen peroxide and superoxide anion. A total of 124 MII oocytes from five statistical replications were used for these assays. For each replication, 7–10 MII oocytes belonging to each group were stained. Oocytes stained with DCFH-DA produced a brilliant green color in the presence of H_2_O_2_ (Fig. 8A). According to Fig. 8B, the Ch-IONPs group (16.68 ± 1.41) has augmented significantly (****P < 0.0001) compared to the control (8.23 ± 0.86) and G-IONPs group (8.26 ± 1.24). However, this increase was insignificant. This finding indicates that G-IONPs synthesized with DPP extract have the best antioxidant properties.

Within the context of DHE analysis, 5–8 MII oocytes were stained for each group and replication. Figure 8C displays the stained oocytes with DHE, resulting in a brilliant red color when exposed to 2-hydroxy ethidium-O2. The Ch-IONPs group showed a significant increase in the amount of superoxide anion (8.30 ± 0.66) compared to the control group (6.21 ± 0.45) (*P < 0.05). No significant statistical difference was observed between G-IONPs (7.02 ± 0.52) and the control group, as well as Ch-IONPs (Fig. 8D).

To examine the ROS triggered by IONPs, the ROS levels in the medium were analyzed individually. Adding l µg/m1 Ch-IONPs to the oocyte maturation medium significantly increased ROS production compared to the control group (*P < 0.05). The difference between G-IONPs and control groups was not significant.

#### In vitro fertilization, development, and blastocyst rates

**Figure 9:**
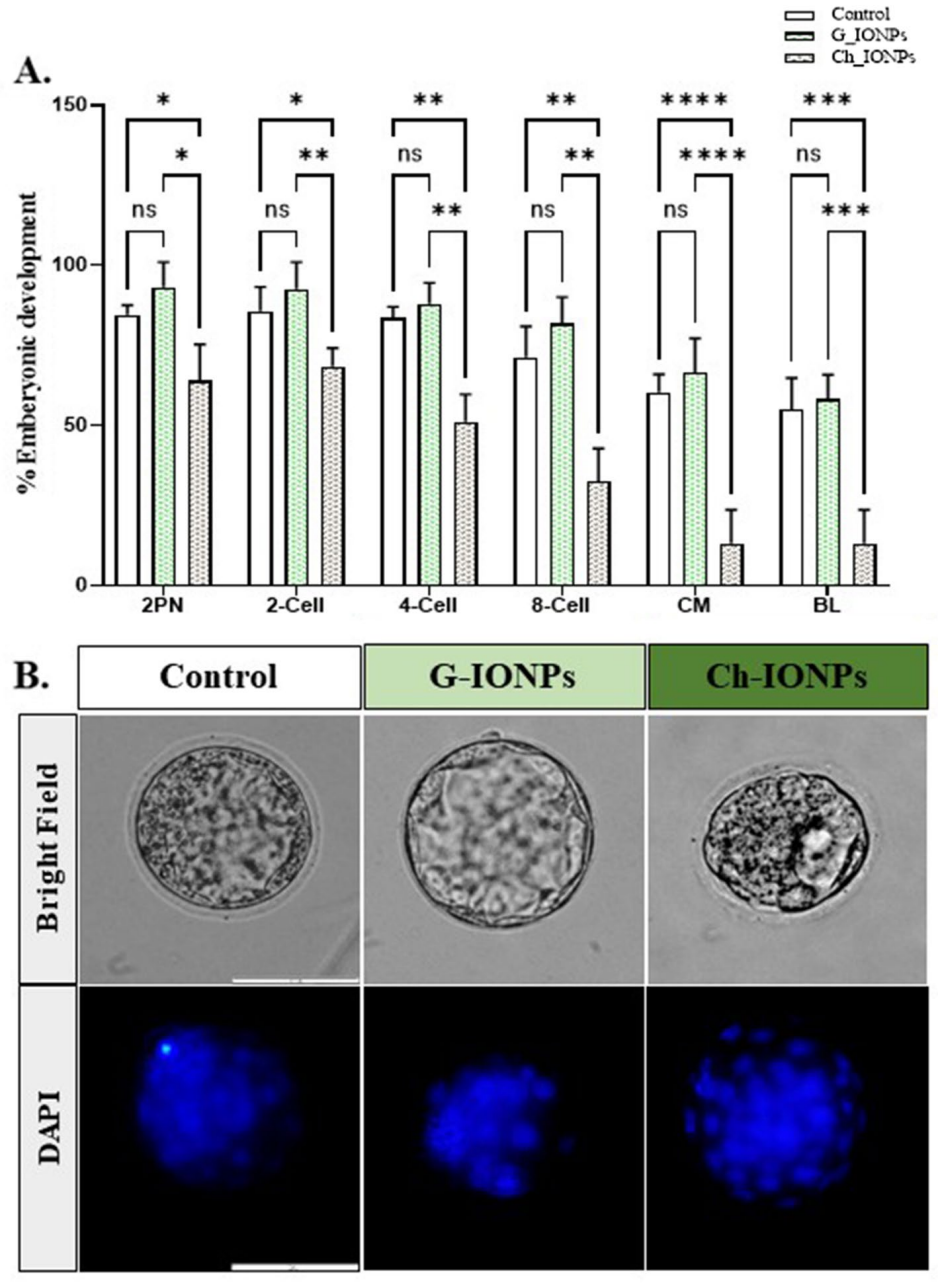
In vitro fertilization. (**A**) Effect of Ch-IONPs and G-IONPs during IVF and embryo development. Each point represents the mean ± SEM of five repeats. P-values are shown at all stage rates (*p < 0.05, **p < 0.01, ***p < 0.001, ****p < 0.0001). (**B**) DAPI staining shows the nucleus formation in the blastomeres of the experimental groups; scale bar 200 μm. *G-IONPs* green synthetized iron oxide nanoparticles, *Ch-IONPs* chemically synthetized iron oxide nanoparticles.

The MII oocytes underwent IVF and subsequent cleavage in four separate statistical replications. The study assessed the rates of development at several stages, namely: fertilized 2PN zygote (2PN/MII), 2-cell (2-cell/2PN), 4-cell (4-cell/2PN), 8-cell (8-cell/2PN), compacted morula (CM/2PN), and blastocyst (BL/2PN). As shown in Fig. 9A, the rate of 2PN in Ch-IONPs (63.89 ± 4.65) declined a lot compared to the control (84.50 ± 1.23) and G-IONPs (93.17 ± 3.18) (*P < 0.05). The rate of 2-cells in Ch-IONPs (68.34 ± 2.36) was lower than in the control group (85.69 ± 3.08) and G-IONPs (92.59 ± 3.42) (*p < 0.05 and **p < 0.01), similar to 2PN. In addition, the rate of 4-cells fell significantly in Ch-IONPs (51.10 ± 3.50) compared to the control (83.61 ± 1.37) and G-IONPs (87.96 ± 2.66) (**p < 0.01). The 8-cell count in Ch-IONPs (32.50 ± 4.17) was significantly lower compared to the control (71.25 ± 83.92) and G-IONPs (81.85 ± 3.35) (**p < 0.01). In addition, the rate of CM in Ch-IONPs (13.06 ± 4.31) was much lower than in the control (60.29 ± 2.28) and G-IONPs (66.48 ± 4.36) groups (****p < 0.0001). The G-IONPs exhibited the highest BL formation (58.15 ± 3.10); however, this difference was not statistically significant compared to the control (54.86 ± 4.05). The value in the Ch-IONPs group (13.06 ± 4.31) considerably decreased compared to the other groups (***p < 0.001).

#### DAPI staining

As shown in Fig. 9B, the release of blue fluorescence signifies the creation of nuclei in blastomeres. The DAPI dye is better attached to DNA in the G-IONPs and control samples. This means that the blastomere nuclei have higher quality. The bright field of Ch-IONPs indicates that these blastocysts are of low quality compared to the control and G-IONPs groups.

## Discussion

The IVM medium contains a higher concentration of free radicals, which can negatively impact the quality of oocytes, harm both the oocytes and the embryos, damage the mitochondria, reduce the fertilization rate, and increase the expression of proteins linked to apoptosis^24^. Hence, it is advisable to use antioxidants to enhance the efficacy of oocyte maturation. In this study, we examined the effectiveness of iron nanoparticles as a cofactor for enzymatic antioxidants. We synthesized the nanoparticles using a green method, specifically by supplementing the oocyte maturation culture medium with DPP aqueous extract. The efficacy of these nanoparticles is compared to those synthesized using a chemical method.

The XRD analysis revealed that the produced IONPs exhibited a sole Fe3O4 phase. The surface charge of NPs is a crucial feature that can impact biocompatibility. The zeta potential analysis revealed that the Ch-IONPs possess a positive charge. Recent studies indicate that Fe_3_O_4_ nanoparticles without a coating and with a positive charge readily traverse the lipid membrane^25^. Nevertheless, the IONPs produced by DPP extract (G-IONPs) exhibited a significant increase in surface charge, which research has linked to improved stability.

Furthermore, the negative charge generated in nanoparticles can decrease their accumulation in cells and mitigate potential adverse effects^26,27^. The FESEM analysis showed that the chemical IONPs have spherical shapes, while the green IONPs take on both spherical and rod shapes when DPP is added. The size range of the IONPs was around 10–30 nm. Decuzzi et al. demonstrated that spherical particles exhibit greater cellular internalization than rod-shaped particles with a higher length-to-radius ratio^28^. Studies have shown that these nanoparticles are less toxic than rod-shaped nanoparticles. The FESEM and zeta potential measurements show that chemical nanoparticles can easily enter the oocyte and may have adverse effects. Additionally, uncoated and small nanoparticles exhibit more toxicity than modified and bulk ones. While G-IONPs can decrease the levels of ROS, the lack of coating on the nanoparticles can reduce their efficacy^25,29^.

According to the DPPH antioxidant assay, the percentage of free radical inhibition by Ch-IONPs is approximately half that of G-IONPs. Consiquently, IONPs coated with DPP can reduce ROS species in the culture medium better than Ch-IONPs. Therefore, the negative charge on the nanoparticles' surface and the presence of antioxidant compounds in the DPP extract coating layer have influenced the decrease in ROS species abundance in the culture medium, compared to bare nanoparticles with a positive charge. Jayanta Kumar Patra and Kwang-Hyun Baek showed in their study that the synthesis of iron oxide nanoparticles with the aqueous extracts of silky corn hairs (Zea mays L.) and outer leaves of Chinese cabbage (Brassica rapa L. subsp. pekinensis) can exhibit strong antioxidant activities in both FeNPs. Other studies also indicated that the green synthesis of iron nanoparticles with other natural extracts creates beneficial effects such as antimicrobial and antioxidant activity, increased stability, a large surface area, high magnetic saturation, and long-term biocompatibility to ensure its use in medical applications^30,31^.

Additionally, this study aimed to investigate the impact of Ch-IONPs and G-IONPs on oocyte maturation parameters. Some research has found that certain chemical nanoparticles affect oocyte maturity indexes. Hong et al. found that silver nanoparticles slow down the IVF process and interfere with mouse oocyte maturation. Data suggests that silver nanoparticles may increase ROS and the percentage of apoptotic cells^32^. In vivo studies have shown that long-term exposure to titanium dioxide (90 consecutive days) in female CD-1 mice (ICR) leads to oxidative stress induction^33^. In addition, in vitro studies have elucidated that α-Fe2O3 nanoparticles can induce ROS and cell death in female mice^34^. Preaubert et al. also proposed that the in vitro fertilization rate of B6-CBA-F1 mouse oocytes in the culture medium containing CeO_2_ attenuated significantly compared to the control, even at low concentrations, which could be due to the oxidative stress induced by CeO_2_ nanoparticles^35^. Free radical production, an increase in ROS, and poor-quality oocytes resulting from incorrect culture methods are the main factors contributing to the failure of IVM and reduced fertility rates in clinical settings^36^.

DPP's antioxidant capabilities, such as catechin, caffeic acid, epicatechin, vanillic acid, coumarin, and vitamins E and C, mitigated these side effects in IONP synthesis^17^. In this study, iron synthesized using a greener approach dramatically decreased free radical levels, which is a factor in accelerating the maturation rate of oocytes. The functional groups of biomolecules in DPP may be the ones responsible for IONPs' negative charge, regeneration, and stability. It can be used as a reducing and capping agent in nanoparticle production. The structural analysis of green synthesized gold and silver nanoparticles demonstrated that the organic components of DPP serve as stabilizing agents and cover^19^.

It proved that the antioxidant potential of DPP can protect the oral mucosa by blocking oxidative free radicals, preventing DNA damage, and neutralizing inflammatory reactions through its bioactive compounds^37^. DPP has antioxidant properties that can improve the survival and growth rates of pre-antral follicles. DPP grain extract may improve the IVM of follicles as a supplement to IVM media in NMRI mice^38^.

Pro-oxidants like ROS are constantly generated in the culture condition as a consequence of cellular metabolic reactions, oxygen concentration, light, oocyte handling, and general physicochemical parameters. In oocyte physiology, ROS are considered signal molecules, and they have recently been found to affect the destabilization of maturation-promoting factors, inducing apoptosis and influencing in vitro oocyte development^39^. Studies suggest that adding iron to the oocyte maturation culture medium could help reduce free radicals. This is because iron acts as a cofactor for the catalase enzyme. In this study, adding IONPs to the oocyte maturation culture medium resulted in a decrease in H_2_O_2_ compared to the control. The findings of Zargari and Khazae et al. corroborate those of our work, which demonstrated that ferrous ions (Fe^3^⁺) reacting with H_2_O_2_ during the Fenton reaction reduce the level of superoxide by converting it into water molecules^40,41^. When comparing the amount of H_2_O_2_ in the oocyte to the control, the addition of Ch-IONPs resulted in an increase in H_2_O_2_ levels compared to the control. Nevertheless, the G-IONPs exhibited a significant antioxidant effect, resulting in a significant decrease in the concentration of H_2_O_2_ in the culture mtedium compared to the control.

The increase in the formation of H_2_O_2_ increases the number of GVBD arrests and inhibits the formation of the first polar body^42^. According to IVM results, the elevation of GVBD in oocytes treated with Ch-IONPs compared to the control was in contrast to G-IONPs; therefore, an interesting point in this study was the potency of IONPs synthesized by the green method to reduce the amount of H_2_O_2_ in the oocyte.

René et al. showed that the antioxidant properties of DPP compounds reduced the amount of superoxide radicals^43^. Molecular oxygen gains one additional electron to generate a superoxide radical. The current study examined the generation of superoxide radicals and found that oocytes treated with Ch-IONPs exhibited a considerable elevation in O_2_^-^ production compared to the control group. However, there was no significant difference in O_2_^-^ production between the oocytes treated with G-IONPs and the control group.

In vitro embryo production involves exposing oocytes to an unnatural environment that leads to excessive and unbalanced levels of intra-oocyte ROS. Deficiency in oocyte maturation and fertilization is a result of oxidative stress that has negative consequences for blastocyst development^42^. For example, an increase in ROS levels during the in vitro production of bovine embryos leads to failed embryonic development^44^. Gao et al. demonstrate that media supplemented with iron exhibited a greater frequency of 8-cell embryos, morulae, and blastocysts. Conversely, a prolonged absence of iron resulted in an elevated count of apoptotic blastomeres^45^. Here, the G-IONPs and control groups showed enhanced fertilization rates and embryonic development compared to the Ch-IONPs group. Furthermore, IONP groups played a crucial role in the viability of the 8-cell embryos, morula, and blastocyst.

Based on our results, we observed an increase in the Mt membrane potential in the G-IONP-treated group, which is one of the best ways to assess the health of an oocyte and the early development of an embryo. Mitochondria are critical for meiotic spindle formation and maintenance before fertilization. Additionally, related to low maturation, fertilization, and embryo development with Ch-IONPs, it must be explained that ROS overproduction, the lower rate of MII oocytes, and attenuation of viability are considered critical reasons.

## Conclusion

The results show that using DPP aqueous extract to make IONPs in a green way is safer and more biocompatible than using chemical nanoparticles. The DPP has enhanced the antioxidant capacity of G-IONPs. During the incubation process, adding G-IONPs to an IVM medium reduced the levels of ROS in the mouse oocytes. Additionally, this led to an improvement in both the survival rate and maturation rate of the oocytes. Chemical and green-produced IONPs enhanced fertilization and embryonic development, but G-IONPs notably increased, specifically in embryonic development. Adding green-synthesized IONPs to an IVM medium sheds new light on human-assisted reproduction.

## Data Availability

All data generated or analyzed during this study are included in this published article.
