# Author Correction: Oocyte maturation, fertilization, and embryo development in vitro by green and chemical iron oxide nanoparticles: a comparative study

**DOI:** 10.1038/s41598-024-75440-y

**Published:** 2024-10-18

**Authors:** Shamim Nejadali Chaleshtari, Elaheh Amini, Farzaneh Baniasadi, Somayeh Tavana, Mohammadreza Ghalamboran

**Affiliations:** 1 Department of Animal Biology, Faculty of Biological Sciences, Kharazmi University, Tehran, Iran; 2 Department of Embryology, Reproductive Biomedicine Research Center, Royan Institute for Reproductive Biomedicine, ACECR, Tehran, Iran; 3 Department of Plants Sciences and Biotechnology, Faculty of Life Sciences and Biotechnology, Shahid Beheshti University, Tehran, Iran

Correction to: *Scientific Reports* 10.1038/s41598-024-65121-1, published online 19 June 2024.

In the original version of this Article, Mohammadreza Ghalamboran was incorrectly affiliated with ‘Plant Sciences and Biotechnology Department, Life Sciences and Biotechnology School, Shahid Beheshti University, Tehran, Iran’. The correct affiliation is listed below.

Department of Plants Sciences and Biotechnology, Faculty of Life Sciences and Biotechnology, Shahid Beheshti University, Tehran, Iran.

The original Article has been corrected.

